# Real-world experience in the management of lipids in patients after an acute coronary syndrome

**DOI:** 10.1016/j.jtumed.2026.05.001

**Published:** 2026-05-21

**Authors:** Omar Shariff, Ruchika Madhotra, Ananya Nair, Ryan Tennyson, Amar Bhagania, Hamid Ullah, Muqeet Enver, Sunil K. Nadar

**Affiliations:** Department of Cardiology, Russells Hall Hospital, Dudley, UK

**Keywords:** إعادة التأهيل القلبي, الدهون, الستاتينات, متلازمة الشريان التاجي الحادة, Acute coronary syndromes, Cardiac rehabilitation, Lipids, Statins

## Abstract

**Objectives:**

After acute coronary syndrome (ACS), the management of lipids in patients forms an essential part of their treatment. Guidelines recommend that these patients should have their lipids checked at least once at 3 months after their index admission, with a target low-density lipoprotein (LDL) level <1.4 mmol/L. In this study, we aimed to assess whether patients had their lipids checked at our institution, and whether they achieved their target LDL levels.

**Methods:**

This study was a retrospective audit of all patients admitted with ACS at our institution over a 12-month period from January to December in 2023.

**Results:**

In total, 303 patients (mean age 67.7 ± 13.5 years; 70% male) were included in the analysis, and 290 of these patients had undergone coronary intervention during admission. Lipids were checked in 237 (77.2%) patients on admission, 109 (36%) at 3 months, 99 (32.3%) at 6 months, and 120 (39.6%) at 1 year. Twenty-two patients (7.3%) were not checked at all (either on admission or follow-up). Lipids were not checked at all on follow-up for 92 patients (30.4%). The LDL level was below the target on admission in only 47 patients. Among the 256 patients who were not below the target, 75 were not checked again. Among those with lipid level checks, 39.4%, 46.4%, and 25.8% were below the target at 3, 6, and 12 months, respectively. The statin doses were not changed for >90% of patients who were not below the target.

**Conclusion:**

At our institution, most patients did not achieve the target LDL level post-ACS. Even when lipids were checked, physician inertia appears to have prevented changes in medications. Thus, steps should be undertaken to ensure better adherence to the guidelines either in the hospital or primary care.

## الملخص

**أهداف البحث:**

يُعد تدبير الدهون لدى المرضى بعد الإصابة بمتلازمة الشريان التاجي الحادة جزءًا أساسيًا من العلاج والوقاية الثانوية. وتوصي الإرشادات بقياس الدهون بعد الدخول الأولي للمستشفى، مع استهداف مستوى كوليسترول البروتين الدهني منخفض الكثافة أقل من 1.4 مليمول/لتر. هدفت هذه المراجعة السريرية إلى تقييم مدى التزام المؤسسة بقياس الدهون لدى المرضى بعد الإصابة بمتلازمة الشريان التاجي الحادة، ومدى تحقيقهم للمستوى المستهدف من كوليسترول البروتين الدهني منخفض الكثافة.

**طرق البحث:**

أُجريت مراجعة سريرية استعادية لجميع المرضى البالغين الذين أُدخلوا إلى المؤسسة بسبب متلازمة الشريان التاجي الحادة، بما في ذلك احتشاء عضلة القلب المصحوب بارتفاع مقطع ST، واحتشاء عضلة القلب غير المصحوب بارتفاع مقطع ST، والذبحة الصدرية غير المستقرة، خلال فترة 12 شهرًا من يناير إلى ديسمبر 2023. تم جمع البيانات من السجلات الإلكترونية، وشملت الخصائص الديموغرافية، وعوامل الخطورة القلبية الوعائية، ونتائج فحوصات الدهون عند الدخول وبعد 3 أشهر و6 أشهر وسنة واحدة من الدخول الأولي، إضافة إلى التغيرات في العلاج الخافض للدهون.

**النتائج:**

شملت الدراسة 303 مرضى، بمتوسط عمر 67.7 ± 13.5 سنة، وكان 70% منهم من الذكور. خضع 290 مريضًا لإعادة تروية تاجية أثناء الدخول الأولي. تم قياس الدهون عند الدخول لدى 237 مريضًا (77.2%)، وبعد 3 أشهر لدى 109 مرضى (36%)، وبعد 6 أشهر لدى 99 مريضًا (32.3%)، وبعد سنة واحدة لدى 120 مريضًا (39.6%). ولم تُقَس الدهون مطلقًا لدى 22 مريضًا (7.3%)، كما لم تُجرَ أي متابعة لمستويات الدهون لدى 92 مريضًا (30.4%). كان 47 مريضًا فقط ضمن المستوى المستهدف لكوليسترول البروتين الدهني منخفض الكثافة عند الدخول. ومن بين 256 مريضًا لم يكونوا ضمن الهدف العلاجي، لم يُعاد قياس الدهون لدى 75 مريضًا. ومن بين المرضى الذين خضعوا لفحص الدهون أثناء المتابعة، حقق 39.4% و46.4% و25.8% المستوى المستهدف بعد 3 أشهر و6 أشهر و12 شهرًا على التوالي. كما أن أكثر من 90% من المرضى الذين لم يحققوا الهدف العلاجي لم تُعدَّل جرعة الستاتين لديهم.

**الاستنتاجات:**

أظهرت هذه المراجعة أن معظم المرضى في هذه المؤسسة لا يحققون المستوى المستهدف لكوليسترول البروتين الدهني منخفض الكثافة بعد الإصابة بمتلازمة الشريان التاجي الحادة. وحتى عند قياس الدهون، يبدو أن الجمود السريري يسهم في عدم تعديل العلاج الدوائي. وتؤكد النتائج الحاجة إلى تحسين الالتزام بالإرشادات من خلال تعزيز تثقيف الأطباء وتطوير آليات متابعة أكثر فعالية داخل المستشفى أو في الرعاية الأولية.

## Introduction

Cardiovascular diseases (CVD), including stroke and ischemic heart disease (IHD), are the leading causes of mortality worldwide.^1^ The global number of CVD-related deaths increased from 12.1 million in 1990 to 18.6 million in 2019, and almost 50% were attributable to IHD.^1^ This increase has significant financial implications, and the costs related to CVD in the United States are expected to more than triple by 2035.^2^

The risk factors that contribute to CVD and IHD are well documented, where they include high cholesterol, smoking, diabetes, obesity, hypertension, and alcohol consumption.^2^, ^3^, ^4^ According to the INTERHEART study, hyperlipidemia was found to be the single most important risk factor for myocardial infarction (MI).^5^ Lipids are carried in the body in the form of lipoproteins, which consist of a mixture of triglycerides, cholesterol, phospholipids, and apoproteins, with varying proportions.^5^^,^^6^ Low-density lipoprotein-cholesterol (LDL-C) is the most dangerous lipoprotein and its accumulation within the vessel intima triggers an inflammatory cascade resulting in atherosclerosis.^6^ The relationship between LDL-C levels and the risk of MI is linear,^4^ and thus LDL-C measurement is currently used to estimate an individual's cardiovascular risk according to both guidelines and in clinical practice.

A reduction in the concentration of LDL-C lowers the risk of CVD, and systematic reviews and meta-analyses have shown that the annual risk of major cardiovascular events (including MI and stroke) is reduced by approximately 20–25% for every 1 mmol/L reduction in LDL-C.^7^^,^^8^ Among those who experience acute coronary syndrome (ACS), there is a 30% risk of recurrence within the first 2 years, and thus lipid-lowering strategies are of even greater importance in this cohort of patients.^9^ It has been suggested that early initiation of lipid-lowering medication with appropriate follow-up can reduce the risk of further cardiovascular events in post-ACS patients.^10^

Statins are the first-line medication option in most guidelines for managing hypercholesterolemia.^9^ Statins act by competitively inhibiting the enzyme HMG-CoA reductase to decrease the synthesis of cholesterol within the liver, leading to the increased expression of LDL-C receptors on the liver's surface and increasing LDL-C uptake from the circulation.^9^ Several randomized controlled trials have demonstrated the benefits of high-intensity statin therapy post-ACS,^11^^,^^12^ with a reduction of up to 50% in LDL-C compared with placebo.^9^^,^^13^ In addition, ezetimibe inhibits intestinal cholesterol absorption and PCSK9 (proprotein convertase subtilisin/kexin 9) inhibitors prevent LDL-C receptor breakdown, and these lipid-lowering medication reduce LDL-C levels by up to 20% and 60%, respectively.^13^ The use of these medications together with statins can result in even greater reductions in LDL-C compared with using any one medication alone.^9^^,^^13^^,^^14^

The most recent 2023 guidelines from the European Society of Cardiology (ESC) for the management of ACS recommend that the LDL-C target level post-ACS should be < 1.4 mmol/L.^15^ Early initiation of high-dose statin therapy (e.g., atorvastatin or rosuvastatin) post-ACS is emphasized, as well as the addition of ezetimibe and/or a PCSK9 inhibitor if the target is not met. The ESC guidelines recommend measuring lipid levels at admission and then 4–6 weeks after changing each medication or dose until the target is reached, whereas the National Institute of Clinical Excellence (NICE) guidelines recommend that lipid levels should be checked at admission and then 2–3 months after treatment has commenced.^16^

Unfortunately, compliance with the ESC guidelines remains poor. A recent large-scale study across Europe demonstrated that 80% of patients failed to achieve their LDL-C target,^17^ thereby highlighting an area of clinical practice in need of significant improvement because it is evident that tight control of lipid levels and adopting a “lower is better” strategy improves outcomes for patients post-ACS.^2^

We audited compliance with the ESC 2019 and 2023 guidelines^13^^,^^15^ within our cardiology department to address three main objectives. First, we assessed how many patients with a confirmed diagnosis of ACS had a lipid profile check during admission, at 3 months, and at 12 months post-ACS. Second, we assessed how many patients reached their target LDL-C level. Third, we determined how many patients had adjustments of their lipid-lowering medications if the target level was not reached.

## Materials and Methods

This retrospective audit involved all adult patients who were admitted with a diagnosis of ACS, including ST-elevation MI (STEMI), non-STEMI (NSTEMI), and unstable angina, to the Dudley group of hospitals NHS trust, UK (which is a publicly funded 700 -bed secondary care district general hospital, serving a population of around 450,000), between the dates of January 1, 2023 and December 31, 2023. We reviewed the electronic records of all eligible patients with these diagnoses and excluded those without an available complete set of records. Patients who were not from our local region and whose follow-up occurred elsewhere were not included in this audit.

Demographic data were recorded, including age, gender, and cardiovascular risk factors. We also recorded the baseline lipids if checked as well as records of their follow-up lipid profile. If patients had more than one admission during the audit period, the first admission was counted as the index admission and other lipid values were taken as follow-up values. Based on the records, we defined time periods of 3 months, 6 months, and 1 year as the time intervals from index admission.

Statistical analyses were performed using SPSS version 21. All data were expressed as a number (percentage), mean (standard deviation, SD), or median (interquartile range). This study was registered as an audit and approval was obtained from our audit department prior to commencing the audit (audit number: CAR/CA/2025-26/05).

## Results

According to our records, 356 patients were admitted with a diagnosis of ACS during the study period, but 53 had incomplete records and were excluded from the study. In total, 303 patients (mean age: 67.7 ± 13.5 years; 212 male (70%), 91 female (30%)) were included in the analysis. Among these patients, 290 had undergone coronary revascularization during index admission. The remaining 13 patients underwent angiography but had coronary atheroma that was not suitable for intervention (minor or diffuse disease). These patients were still considered to be NSTEMI due to plaque rupture, and thus included in the analysis. The baseline risk factors are shown in Table 1. In terms of lipid profile measurements, 234 patients (77%) had their lipids checked on admission, 109 patients (36%) at 3 months, 99 patients (32.6%) at 6 months, and 120 (40%) at 12 months (Table 1).

**Table 1:** Baseline demographic data for the patient population. PCI/CABG: percutaneous coronary intervention/coronary artery bypass grafting.

	Number (percentage) n = 303
Age	67.7 ± 13.5 years
Gender (male:female)	212 (70%):91 (30%)
Diabetes	91 (30%)
Hypertension	166 (54.7%)
Hyperlipidemia	97 (32%)
Smoking	84 (27.7%)
Previous PCI/CABG	79 (26.1%)
Number who had lipids checked at	
3 months	109 (36%)
6 months	99 (32.3%)
1 year	120 (39.6%)
Number of times lipids checked over 12 months	
Never	92 (30.4%)
Once	112 (36.9%)
Twice	82 (27.1%)
Three times	17 (5.6%)

Twenty-two patients (7%) did not have their lipid profiles checked at all (either at admission or on follow-up), and 70 patients (23%) had their lipids checked only on admission with no follow-up checks. Among those who had follow-up lipid checks, 112 patients were checked once (36.9%), 82 were checked twice (27.1%), and 17 were checked three times (5.6%) (Table 1).

**Figure 1:**
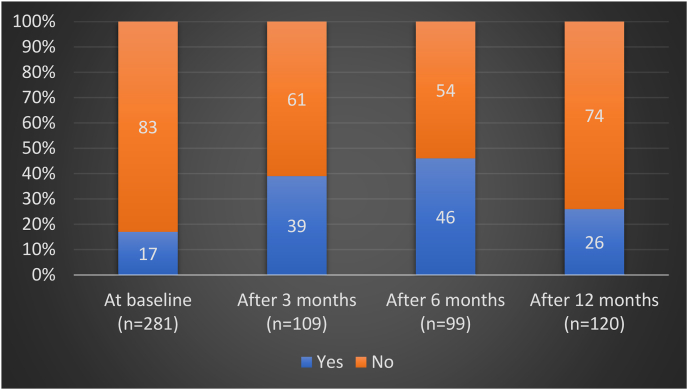
Bar chart showing percentages of patients who reached target of LDL <1.4 mmol/L at baseline and on follow-up.

Based on the ESC guidelines for lipid management, using an LDL level of 1.4 mmol/L as the cut-off, 47 out of 281 patients (17%) met their LDL target at baseline. Subsequently, 43 of 109 patients (39%) met their LDL target at 3 months, 46 of 99 patients (46%) at 6 months, and 31 out of 120 patients (26%) at 12 months (Figure 1).

Assessment of lipid-lowering therapies showed that 148 out of 303 patients were already on a statin on admission. Among these 148 patients, there was no change in the dose for 62 patients, the dose increased for 54, the statin was changed for 27, and another lipid-lowering agent was added for five. Among the remaining 132 patients who were not on a statin on admission, 126 were started on a statin prior to discharge. No documentation was available for 29 patients about whether they were on statin therapy prior to admission or if they started on statin therapy.

After three months, among the 66 patients who were not at their target LDL (i.e., <1.4 mmol/L), the statin dose was increased for five patients, the statin was changed for one, the statin dose was reduced for two, and a new lipid-lowering agent was added for four. There were no changes in the medications for the remaining 54 patients (81.8%). After 6 months, among 51 patients who were not at their target LDL, the statin was changed for one, the statin dose was reduced for one, a new agent was added for three, and the statin dose was increased for none. There were no changes in the medications for the remaining 46 patients (90.1%). After 12 months, among 87 patients who were not at their target LDL, the statin dose was increased for one patient, the statin was changed for three, the statin dose was reduced for three, and a new agent was added for six. There were no changes for the remaining 74 patients (85%). These findings are summarized in Table 2.

**Table 2:** Number of patients with lipid-lowering medication adjustments at 3, 6, and 12 months, and whether they reached the target LDL level. Figures represent numbers (%).

	3 months (n = 109)		6 months (n = 99)		12 months (n = 120)	
	Not at target LDL (n = 66)	On target LDL (n = 43)	Not at target LDL (n = 53)	At target LDL (n = 46)	Not at target LDL (n = 88)	At target LDL (n = 32)
No change in dose	54 (84.8%)	41 (95.3%)	48 (90.5%)	42 (91.3%)	75 (85.2%)	29 (90.6%)
Increased dose	5 (7.5%)	0	0	1 (2.1%)	1 (1.1%)	0
Changed statin	1 (1.5%)	1 (2.3%)	1 (1.8%)	0	3 (3.4%)	0
Decreased dose	2 (3%)	0	1 (1.8%)	3 (6.5%)	3 (3.4%)	3 (9.4%)
New drug added	4 (6%)	1 (2.3%)	3 (5.6%)	0	6 (6.8%)	0

Despite the less than optimal dosage adjustments, significant changes were found in the mean LDL value over time. The mean LDL value at admission was 2.52 ± 1.18 mmol/L. On follow-up at 3 months, 6 months, and 1 year, the mean LDL values were 1.67 ± 0.75 mmol/L, 1.59 ± 0.75 mmol/L, and 1.83 ± 0.66 mmol/L, respectively. According to paired analysis between the values at baseline and after 1 year (where paired samples were available; n = 120), the mean ± standard deviation were 2.67 ± 1.27 mmol/L at baseline and 1.78 ± 0.63 mmol/L at 1 year (*p* < 0.001).

## Discussion

The results obtained in this audit demonstrate that current compliance with ESC guidelines for the management of lipids post-ACS was suboptimal across all of our objectives. Not all patients admitted with an ACS had their lipids checked on admission and even less had it checked on follow-up. Even when the lipids were checked, the lipid-lowering agent dosage was not altered, and thus less than one-third achieved the target LDL level after 1 year.

Unfortunately, the poor achievement of lipid targets post-ACS is not limited to only our center or even the United Kingdom. Our results are mirrored by a number of recent observational studies in Europe, the United States, and India, which demonstrate that achievement of LDL-C targets post-ACS remains a global issue.^2^ According to a meta-analysis of seven studies across Europe in 2023, only 12.1% of patients achieved an LDL-C target of <1.4 mmol/L post-ACS.^18^ Similarly, a multicenter trial in India demonstrated that only 20.9% of post-ACS patients achieved the same LDL-C target after 1 year.^19^ Thus, the findings obtained from several multi-center studies and the results produced in the present audit of our own local institution highlight the disparity between clear evidence regarding the benefits of lowering LDL-C levels post-ACS, and the real-world implementation of the ESC guidelines.

Several potential reasons may explain why compliance with these guidelines is suboptimal, particularly the notion of clinical inertia, which occurs when healthcare professionals fail to act upon the results obtained for patients despite their knowledge. In the present study, clinicians might not have intensified or adjusted lipid-lowering treatments to meet LDL-C targets due to a combination of factors, including limited appointment time, overestimation of a clinician's own adherence to guidelines, frequent changes to guidelines, concerns regarding side effects of very low LDL-C levels, and reluctance to challenge patients who wished to reduce LDL-C through lifestyle changes or those concerned about side effects.^20^ A recent study by Faggiano et al. suggested that the use of applications could help to reduce physician inertia, where they conducted Monte Carlo simulations with more than 10,000 iterations and predicted good compliance with the guidelines.^21^

Results obtained from the PALM registry showed that 59% of patients who were not on a statin were not offered one, highlighting the existence of clinical inertia in real-world practice.^22^ This issue was also prevalent in our own study where clinicians did not adjust lipid-lowering therapy in up to 87% of patients, despite them not achieving the LDL-C target. However, there is concern regarding very low LDL-C levels, although evidence suggests that there are no increases in the frequency of adverse effects such as diabetes, stroke, or cognitive impairment under very low LDL-C levels.^2^ One method of overcoming clinical inertia would be integrating the guidelines and treatment algorithms into a clinical decision support system, which could prompt clinicians during a consultation to adjust medications according to the LDL-C target.^2^^,^^20^

Second, patient adherence to lipid-lowering therapy remains a barrier to achieving LDL-C targets post-ACS. The most common reason reported in the PALM registry for discontinuation of statin therapy in secondary prevention was side effects in 60.3% of patients.^22^ Other reasons included the perception that medications were no longer needed (13.5%), preference for natural remedies (6.3%), cost of medication (7.2%), reluctance to take medication every day (4.6%), suggestions from relatives or friends (4.2%), and the lack of noticeable improvement (4.2%).

Negative public perception of statins has arisen due to fear of muscle-related symptoms, although true statin-induced muscle damage (such as myopathy or rhabdomyolysis) is a rare occurrence.^13^ Studies have shown that patient-reported side effects from statins increased when patients were not blinded to the treatment they received, highlighting the “nocebo effect” where negative expectations of a treatment lead to an increase in reported side effects.^2^^,^^13^ Statin-related muscle side effects can be difficult to manage, although changing the statin or reducing the dosage can help to improve compliance with statin therapy.^13^ Furthermore, treatment with other non-statin agents such as bempedoic acid is a good alternative in statin-intolerant patients because it is generally well tolerated with rare side effects, and is effective in reducing LDL-C.^23^

Other reasons for poor adherence can be addressed through patient education about the importance of lowering LDL-C, the intended benefit of lipid-lowering therapy in preventing a further episode of ACS, and exploring the concerns of patients regarding potential side effects. We did not measure adherence to treatment during our audit due to its retrospective nature and the difficulty collecting such data, but this is an important issue to consider following intervention and re-audit.

Despite the aforementioned limitations achieving target LDL-C levels, the “Jena auf Ziel” study demonstrated that achieving LDL-C targets post-ACS is possible with early combination therapy.^24^ Similarly, the use of good rehabilitation programs was demonstrated to help achieve LDL targets post-ACS.^25^ All patients were started on a high-intensity statin and ezetimibe prior to discharge, and treatment was escalated with either bempedoic acid or PCSK9 inhibitors if targets were not met after 4–6 weeks. All patients in the study achieved an LDL-C level <1.4 mmol/L within 12 months of the index event, and with no significant side effects. This early combination strategy is supported by the 2023 ESC guidelines, which suggest that dual therapy with statins and ezetimibe should be considered during ACS hospitalization for those who are already on maximum dose statin therapy, or those who are not on statin therapy but have high LDL-C levels that indicate targets are unlikely to be reached with statins alone.^15^

This early combination approach is not yet widely implemented, but should be considered as part of an overall strategy for achieving LDL-C targets post-ACS. Furthermore, a recent review article suggested that implementing dedicated post-ACS clinics with multidisciplinary team inputs could improve lipid management post-ACS.^26^ Both cardiology pharmacists and clinical nurse specialists play pivotal roles in patient education and improving medication compliance. Indeed, in a nurse-led post-ACS clinic, 64% of patients met an LDL-C target of <1.4 mmol/L after only 4 months.^27^ Automating referral systems on discharge to such clinics as well as integrating virtual follow-up methods for patients who are unable to attend face-to-face would also help to improve compliance and prevent loss to follow-up.^26^

One of the main limitations of our audit was that we did not study the reasons for noncompliance with the guidelines. We found poor compliance but are not able to suggest possible explanations. Thus, in future research, it would be useful to contact the physicians to enquire about barriers to better lipid control. The present study was a retrospective audit, and thus it had inherent limitations such as possible poor record keeping and dependence on the quality of electronic records. In some cases, the lipid-lowering drug dosage might not have been increased due to intolerance but not documented. However, our only information was that documented in the patient records. A high proportion of patients did not receive follow-up or lacked data, which could have affected the validity of the results. Despite this possible limitation, our study highlights the need for continued physician education and giving them reminders to improve lipid management post-ACS, and it is even possible that the results obtained after including the missing data may have indicated even worse lipid management.

## Conclusion

Lipid management remains an essential but poorly executed aspect of post-ACS care. The current guidelines stress the importance of strict lipid control but this was not achieved in the real-world setting at our institution. Strategies to improve lipid management following ACS, including more education for physicians and health care workers, should be implemented in order to achieve better patient outcomes.

## Authors’ statement

The authors declare that all persons designated as authors qualify for authorship and have checked the article for plagiarism. If plagiarism is detected, all authors will be held equally responsible and will bear the resulting sanctions imposed by the journal thereafter.

## Ethical approval

This study was registered as an audit and approval was obtained from our audit department prior to commencement of the audit (audit number: CAR/CA/2025-26/05).

## Source of funding

This research did not receive any specific grant from funding agencies in the public, commercial, or not-for-profit sectors.

## Conflict of interest

All authors declare no conflict of interest in the production of this manuscript and study
